# A Physiological-Based Model for Simulating the Bioavailability and Kinetics of Sulforaphane from Broccoli Products

**DOI:** 10.3390/foods10112761

**Published:** 2021-11-10

**Authors:** Quchat Shekarri, Matthijs Dekker

**Affiliations:** Food Quality and Design Group, Wageningen University and Research, P.O. Box 17, 6700 AA Wageningen, The Netherlands; quchat.shekarri@wur.nl

**Keywords:** physiological-based model, sulforaphane, glucoraphanin, compartmental model, broccoli, bioavailability, myrosinase, parameter estimation

## Abstract

There are no known physiological-based digestion models that depict glucoraphanin (GR) to sulforaphane (SR) conversion and subsequent absorption. The aim of this research was to make a physiological-based digestion model that includes SR formation, both by endogenous myrosinase and gut bacterial enzymes, and to simulate the SR bioavailability. An 18-compartment model (mouth, two stomach, seven small intestine, seven large intestine, and blood compartments) describing transit, reactions and absorption was made. The model, consisting of differential equations, was fit to data from a human intervention study using Mathwork’s Simulink and Matlab software. SR urine metabolite data from participants who consumed different broccoli products were used to estimate several model parameters and validate the model. The products had high, medium, low, and zero myrosinase content. The model’s predicted values fit the experimental values very well. Parity plots showed that the predicted values closely matched experimental values for the high (*r*^2^ = 0.95), and low (*r*^2^ = 0.93) products, but less so for the medium (*r*^2^ = 0.85) and zero (*r*^2^ = 0.78) myrosinase products. This is the first physiological-based model to depict the unique bioconversion processes of bioactive SR from broccoli. This model represents a preliminary step in creating a predictive model for the biological effect of SR, which can be used in the growing field of personalized nutrition.

## 1. Introduction

To determine the bioavailability of bioactive compounds in foods, it is important to know its composition, structure, how it interacts with other food components, and its fate in the human body after being ingested. Isothiocyanates (ITC) are formed from precursors, glucosinolates (GL), which are found in broccoli and other types of brassica vegetables [1]. Numerous studies investigated the health effects of some ITCs. One such ITC is called sulforaphane (SR), derived from the GL glucoraphanin (GR). Sulforaphane is known to reduce the risk of cancer, and has cardiovascular and central nervous system protection benefits [1,2].

**Figure 1:**
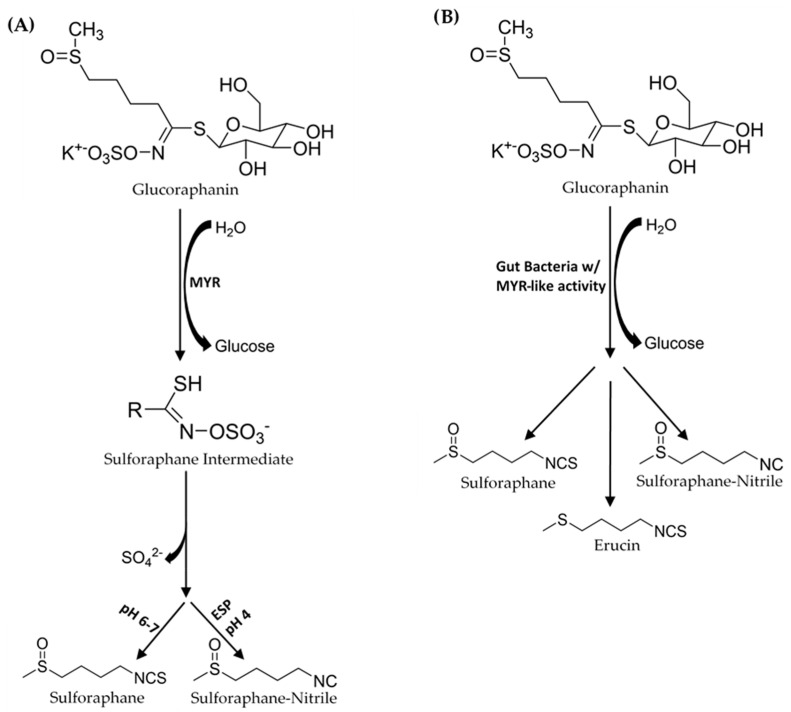
(**A**) In the mouth, myrosinase (MYR) converts glucoraphanin (GR) to an SR-O-sulfated thiohydroximate intermediate. Depending on pH conditions sulforaphane (SR) forms. SR-nitrile formation is preferred at low pH in the presence of epithiospecifier protein (ESP). (**B**) In the gut GR is converted to SR, SR-nitrile, and erucin by gut bacteria.

Sulforaphane’s health benefits have resulted in studies that investigated the physiological mechanisms involved in digesting plants that contain SR, and SR’s absorption, metabolism, and excretion [3,4]. Glucoraphanin is converted to SR by plant endogenous myrosinase (MYR), a β-thioglucosidase hydrolase that catalyzes the removal of glucose to form an O-sulfated thiohydroximate intermediate (Figure 1). GLs and MYR are stored in separate compartments in the broccoli plant cells. Cell structure disruption, from processing (chopping, blanching, powdering etc.), mastication, or plant bruising, is required before MYR can bind GL to facilitate ITC formation [5]. Gut bacteria in the colon also have the capability to facilitate this conversion from GR to SR [6,7,8,9,10,11,12,13,14]. After ingestion, and transfer through the stomach, SR is absorbed in the intestinal tract into the blood, then distributed to various organs before it is eliminated from the body, mainly via renal excretion [15].

The bioavailability of SR is described as the fraction of the amount of SR that is ingested and/or formed in the body that reaches systematic circulation [3]. Related to bioavailability is bioaccessibility, which is the fraction of a compound that is released from the food and that reaches the absorption site. In the context of GLs, MYR and ITCs, the bioaccessible ITC is the fraction of ITCs released from the food matrix [3] or the fraction of GLs transformed to ITCs and released in the body. Bioaccessibility of ITCs is affected by the plant’s inherent GL content (which varies from 47 to 806 mg/100 g fresh weight of broccoli [1]), processing, the food matrix [16,17,18], and the digestion [17,19,20].

The bioaccessibility of ITC could increase or decrease depending on the type of processing. Chopping, blending, powdering are particle size reduction methods that rupture the plant tissue and allow for MYR and GLs to diffuse out and bind to each other [5]. Heating affects epithiospecifier proteins (ESP), MYR, and GL content. ESPs are responsible for the conversion of GLs to nitriles [1] and are less heat stable than MYR. Their inactivation allows for the preferential formation of ITCs [21,22]. Any type of prolonged high temperature heating, however, may cause MYR denaturation [5,19,21] and GL thermal degradation [21]. Freeze drying has been shown to retain MYR and GLs [3]. Prior to freeze drying, microwave cooking at adequate power inputs, inactivates ESP while preserving MYR activity which increases bioaccessibility.

SR formation occurs mostly in two organs: the mouth during mastication and in the gut by the microbiota. Research shows that there are differences between individuals in oral processing of foods [23]. Sarvan et al. [20] investigated, in-vivo, the effect of steaming and chewing time on the bioaccessibility of SR and SR-Nitrile, the GR breakdown products after chewing. Results showed that longer chewing times of broccoli with active MYR led to more GR hydrolysis. Compared to raw broccoli, or broccoli steamed for shorter periods, chewing broccoli steamed for 2 min provided the highest amount of SR. Broccoli steamed for 3 min provided the least amount of SR [20]. The effect of chewing on bioavailability was demonstrated by Shapiro et al. [24] who measured the difference in the amount of ITC metabolites excreted from urine when broccoli sprouts were swallowed whole or chewed thoroughly. They found that chewing increased the amount of urine metabolites by 1.5 times.

In broccoli products with inactivated MYR due to prolonged heating, the gut conversion processes of GR to SR and other degradation products become important. The capability of an individual’s gut microbiome to convert GR to SR will depend on the types of microbes, their quantities, and how effective their different mechanisms for bioconversion are. Gut bacteria convert GLs to other compounds besides ITCs (Figure 1). Saha et al. [6] used a batch fermentation model with human gut bacteria to demonstrate that gut bacteria is capable of converting GR to SR, SR-nitrile, erucin, and erucin-nitrile. They also showed that the formation of erucin is preceded by the microbial conversion of GR to glucoerucin [6]. Consequently, the bioconversion of GL to non-ITC breakdown products reduces the bioavailability of ITCs.

Capturing the essence of the physiological processes for SR mathematically so that its biological effect can be simulated and predicted, is known as physiological-based modelling. This is an approach that considers the physiological basis of a bioactive compound’s interaction with the human body before mathematical concepts are applied. Physiological-based models vary in terms of the number of physiological aspects (i.e., biological mechanisms, organs) considered. Some models only look at the gastrointestinal tract while others consider the whole body [25].

Various types of compartmental model have been described of which the most basic is the compartmental absorption and transit (CAT) model. In the CAT model, the small intestine is divided into a series of compartments and assumes linear transfer kinetics, passive absorption kinetics and well mixed compartments with uniform concentration [26,27]. The transit and absorption of a drug or food component is depicted by the following equation,

(1) $$\frac{dM_{n}}{dt}=K_{t}M_{n-1}-K_{t}M_{n}-K_{a}M_{n},\;n=1,2,\;\ldots 7$$

where *n* is the number of compartments, *M* is the amount or concentration of the component in the *n*th compartment, *K_t_* is the transit rate constant between compartments, and *K_a_* is the absorption rate constant of the component into the blood.

Based on the CAT model, the advanced compartmental absorption and transit (ACAT) model was developed to include more details. The ACAT compartmentalizes the stomach and large intestine so that gastric emptying and absorption from the large intestine can be considered. In addition to linear kinetics and passive absorption, the model considers non-linear kinetics due to protein binding, liver metabolism, or active transport and physiochemical factors such as particle size, solubility, density, and permeability [28].

Most physiological-based modeling research available are for pharmaceutical drugs. There are few studies that are related to food components and food products, and even fewer studies for modeling broccoli compounds. Punt et al. [29,30] made whole body eight-compartment models to predict the bioactivation and detoxification of herb estragole in humans and rats. Le Feunteun et al. [31] made a five-compartment model that focused on the digestion of mini-pigs to study the effect of product matrices on the digestion of milk proteins. Strathe et al. [32] also made a model with four main compartments and 38 sub-compartments to study the digestion and absorption of macro-nutrients in growing pigs. Moxon et al. [33] made a two-compartment model to investigate the effect of gastric emptying, luminal viscosity and hydrolysis rate on the rate of glucose absorption.

At the time of writing this article one study was found about the physiological-based modeling of SR from broccoli. Li et al. [34] investigated the kinetics and distribution of sulforaphane in the tissues of mice using a physiological-based model, where the whole body was divided into eight compartments. The mice ingested fresh, steamed, and MYR treated steamed broccoli sprout powders. The difference in kinetics and distribution in the tissues between the three different products were compared. The model did not include SR absorption mechanisms, and it did not include GR to SR conversion processes in the mouth, via myrosinase, and in the gut, via microbes. Also, the study did not extrapolate their results from mice to humans. The conversion of GR in the gut and mouth are important process that affect bioavailability. Therefore, a physiological-based model that considers these processes is needed.

The objective of this study was to make a physiological-based model that describes the kinetics and bioavailability of isothiocyanates from broccoli and to evaluate how the derived parameters are impacted by inter-individual variation. The model is validated against urine excretion sulforaphane data from a previous 2014 Wageningen University in-vivo research study by Oliviero et al. [35]. In this study, the effect of residual myrosinase activity on ITC formation, bioavailability, and excretion kinetics was investigated after 15 test subjects (apparently healthy human volunteers, aged 26–50 years, body mass index 21 ± 2 kg/m^2^, six men and nine women, 13 Caucasian, two Asian, and one Latin American), consumed five broccoli products with different levels of myrosinase activity obtained by different levels of microwave heating.

## 2. Materials and Methods

### 2.1. Pre-Modeling Data Processing

Participant raw data (measured SR urine conjugate excretion rates) from the Oliviero et al. [35] study was preprocessed for use in Matlab. The data were the time (minutes) and sulforaphane (SR) excretion rate (µmol/min). The technique used to measure SR urine conjugates, solid phase extraction-HPLC-MS/MS [35,36,37], is associated with experimental error that was quantified by Vermeulen et al. [37]. The relative standard deviation 12, 6, 3% for 1.04, 10.5, and 313 µM SR, respectively, in urine, was used to derive the following exponential equation that helped estimate the experimental error of each data point.

(2) $$y=0.11505x^{-0.24}$$

The relative error ratio is y, and the concentration of SR is *x*. The experimental errors were plotted as error bars on the data points for the model fittings.

### 2.2. Model Description and Assumptions

**Figure 2:**
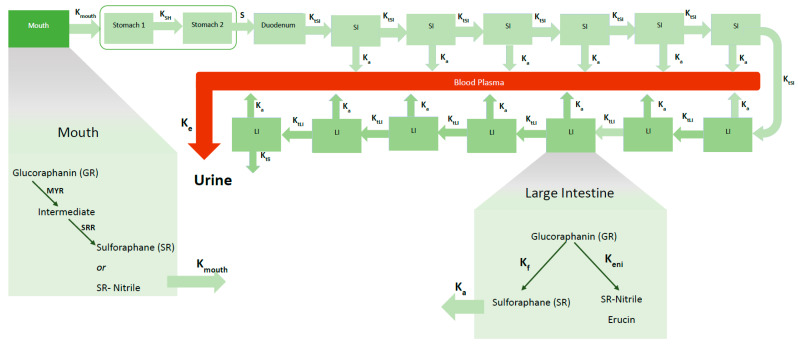
Schematic diagram of model with one mouth compartment, two stomach compartments, seven small intestine (SI) compartments and seven large intestine (LI) compartments, and one blood compartment. The conversion of glucoraphanin (GR) to sulforaphane (SR) and SR-nitrile and erucin in the mouth and large intestine is depicted. Shown are the transit rate constants between the mouth and stomach (K_mouth_), between the two stomach compartments (K_SH_), between the stomach and duodenum (S), between the small intestines (K_tSI_), between the large intestines (K_tLI_). The absorption of SR from the SI and LI into the blood is represented by the absorption rate constant (K_a_). Elimination of SR and SR-conjugates from the blood to urine is represented by the rate constant, K_e_. During mastication, GR is converted to an intermediate by myrosinase (MYR) and the subsequently converted to SR or SR-nitrile based on a conversion ratio (SRR). The gut microbial conversion of GR to SR and SR-nitrile/erucin is represented by the rate constants K_f_ and K_eni_, respectively.

The model (Figure 2) focuses on the processes involved in the gastro-intestinal transit of glucoraphanin (GR) and sulforaphane (SR). Similar to an advanced compartmental absorption and transit (ACAT) model, it includes the stomach, seven compartments of the small intestine [28], the colon, and a blood compartment for systemic circulation. Unlike an ACAT, the colon, was divided into seven compartments, the stomach into two compartments [31]. A mouth compartment, which is typically not in physiological-based models, was included. As a result of this the full model contains 18 compartments.

The products consumed in the intervention study were portions of 5 g of each broccoli product with 90 mL of water at 40 °C, and with 30 g of raisin bun and water ad libitum. During mastication, myrosinase (MYR) and GRs released from the cell structures react to form an O-sulfated thiohydroximate intermediate, which then immediately converts to SR or SR-nitrile. The amount of the intermediate that is converted to SR versus SR-nitrile is a ratio that is subject to change depending on the individual’s chewing pattern and broccoli product. In the mouth, it is assumed that SR-nitrile is the only non-ITC compound formed. Mastication time (30 s) and saliva flow rates (0.033 mL/s) [38] are assumed to be the same for all participants. The volume of the mouth compartment is the product plus the saliva excretions, 0.096 L.

Swallowing transfers the bolus to the stomach. The first stomach compartment accounts for the disintegration of food particles that are too big to pass through the pyloric sphincter valve leading into the small intestine. Food broken down sufficiently, and mixed with gastric fluids in the first compartment, is moved to the second stomach compartment where it mixes with more gastric juices before emptying into the duodenum. Gastric emptying of solid foods has been described as having a biphasic nature due to the time required for enzymatic and mechanical disintegration before emptying into the intestines [39]. Any MYR is assumed to be deactivated irreversibly in the stomach due to the low pH of the gastric fluids [19,40]. It is also assumed that GLs and ITCs are not absorbed in the stomach. Transit from second stomach compartment to the duodenum depends on the stomach emptying time (30 min) which is assumed to be the same for all participants since the size of the meal is the same. Based on the meal size, the volume of both stomach compartments together is 0.2 L, of which 0.05 L is the volume of the first stomach compartment (Table 1).

The chyme is mixed with duodenal secretions in this first compartment of the small intestine (SI). Due to the differences in the intestinal lining of duodenum (less villi/area for absorption) compared to the rest of the small intestine, less nutrients are absorbed in the duodenum; for simplicity, it is assumed there is no ITC absorption. In the remaining six SI compartments, SR is absorbed into the blood as both GR and SR are transferred from one compartment to the next.

The large intestine is divided into seven compartments where the following processes takes place: formation of SR, nitriles, and erucin by gut bacteria, absorption of SR into the blood, and transit of compounds from one compartment to the next. It is assumed that GRs are not absorbed from both the small and large intestines. Movement of chyme in the intestines are in the forward (towards the rectum) direction. Backward movements are known to occur and are represented by the fact that the compartments are assumed to be well mixed.

ITC is absorbed into the blood plasma, which is represented by one compartment that is presumed to be the same volume between all participants (5 L). Absorption of ITCs across the intestinal wall occurs passively by diffusion and is described according to Fick’s Law of Diffusion [41]. ITC metabolites are eliminated from systemic blood circulation via glomerular filtration. No other elimination processes (sweat, defecation, respiration) are considered.

All compartment volumes (Table 1) are assumed to stay constant and the concentration of compounds (GR and SR) per compartment is uniform.

**Table 1:** Parameters values used in the model for the broccoli products, oral GR conversion processes, gastro-intestinal processes, gut GR conversion processes. Parameter values for the broccoli products MYR, Cgl_0_ (initial glucosinolate concentration), and ITC_0_ (initial sulforaphane concentration) are separated by product (HighBP, HighBF, MedBF, LowBF, NoBF, each of these products have different MYR content due to different levels of microwave heating). Parameters designated as ‘Estimated’ were used in model fittings.

Broccoli Products Composition				
Product	MYR Myrosinase Content (mg MYR/mg Broccoli)	$Cgl_{0}$ Initial GR Concentration (µM)	$ITC_{0}$ Initial SR Concentration (µM)	Reference
HighBP	3.49 × 10^−2^	383.3	354.2	[35,42]
HighBF	3.49 × 10^−2^	621.9	115.6	[35,42]
MedBF	6.53 × 10^−3^	667.7	69.8 ^†^	[35,42]
LowBF	5.63 × 10^−4^	708.3	29.2 ^†^	[35,42]
NoBF	1.13 × 10^−5^	734.4	3.1	[35,42]
Oral GR Conversion				
$V_{max}$	MMSI *V_max_* for glucoraphanin		2070 µmol/min	[42]
$K_{m}$	MMSI *K_m_* for glucoraphanin		110.2 µM	[42]
$K_{i}$	MMSI *K_i_* for glucoraphanin		893.0 µM	[42]
BR	Amount of broccoli in broccoli product		5000 mg	[35]
SRR	Fraction of sulforaphane converted from GR in mouth		Estimated	
Gastro-Intestinal				
$k_{Mouth}$	Mouth to Stomach rate constant		30 min^−1^ (60 min^−1^ to 1 min^−1^) *	[43,44]
St	Stomach emptying time		30 min	
S	Gastric rate constant from 2nd stomach to duodenum		S = −ln(0.05)/St	[45]
$k_{SH}$	Rate constant from 1st to 2nd stomach compartment		Estimated **	
$k_{tSI}$	Small Intestine transit rate constant		Estimated **	
$k_{tLI}$	Large Intestine transit rate constant		Estimated **	
$k_{a}$	Absorption rate constant		0.180 min^−1^	[41]
$k_{e}$	Elimination rate constant of ITC and ITC conjugates from blood		Estimated **	
$N_{SI}$	Number of SI compartments (excluding duodenum)		6	[26]
$N_{LI}$	Number of LI compartments		7	[26]
$V_{Mouth}$	Product + Saliva		0.096 L (0.095–0.098 L) *	[35,38]
	Stomach: $V_{Mouth}$+ Raisin bun + gastric secretions		0.2 L (0.167–0.253 L) *	[35,46]
$V_{Stomach\;1}$	Stomach 1		0.05 L	
$V_{Stomach\;2}$	Stomach 2		0.15 L	
$V_{duodenum}$	$V_{stomach\;2}$+ duodenal secretions		0.2 L (0.246–0.332 L) *	[46]
$V_{SI\#}$	SI volume (excluding duodenum)		1.5 L (0.638–1.963 L) *	[47]
	$V_{SI}/N_{SI}\;$= SI compartment volume		0.25 L	
$V_{LI\#}$	LI volume		3.4 L (3.347–3.492 L) *	[47]
	$V_{LI}/N_{LI}$= LI compartment volume		0.5 L	
$V_{17}$	Blood volume of adult		5 L (4–6 L) *	[48]
Gut GR Conversion				
$k_{f}$	Microbial ITC formation rate constant		Estimated	
$k_{eni}$	GR to erucin and nitriles		Estimated	

* Ranges for the parameters were determined based on literature. ** These values were estimated for each individual participant based on the model fit of the experimental values. ^†^ ITC_0_ values used in final fittings for MedBF and LowBF were approximately 3.4 and 9.1%, respectively, of their values in this table due to poor fit results using the original values.

### 2.3. Compartmental Mathematics

The enzymatic reaction of GL and MYR to form the O-sulfated thiohydroximate intermediate in the mouth is characterized by a Michaelis–Menten equation that accounts for enzyme inhibition. The intermediate instantly reacts to form ITC or ITC-nitrile, therefore the change in GL concentration is negatively proportional to the Michaelis–Menten equation.

(3) $$\frac{dC_{GL}}{dt}=-\left(\frac{V_{max}\times MYR\times BR\times C_{GL\;Mouth}}{K_{m}+C_{GL\;Mouth}+\frac{C_{GL\;Mouth}{}^{2}}{K_{i}}}\right)$$

*MYR* is the estimated mg of myrosinase per one mg of dried broccoli. Details on how *MYR* was estimated is found in Appendix A Part I. *BR* is the amount of dried broccoli (5 g) consumed by the participants. The maximum rate, *V_max_* (µM/min*mg *MYR*), the Michaelis constant, *K_m_* (µM), and the inhibition constant, *K_i_* (µM) were derived using glucoraphanin data from Roman et al.’s [42] MYR kinetic study. Details on the derivation of these variables can be seen in Appendix A Part II.

The amounts of ITC and nitriles formed is expressed as a fraction of the amount of intermediate (Equations (4) and (5)).

(4) $$\frac{dC_{ITC\;Mouth}}{dt}=SRR\times \frac{dC_{GL}}{dt}$$

and

(5) $$\frac{dC_{Nitrile\;Mouth}}{dt}=\left(1-SRR\right)\times \frac{dC_{GL}}{dt}$$

*SRR* is the fraction of hydrolyzed *GR* converted to *SR* in the mouth. The remaining, 1 − *SRR*, is converted to nitriles.

Transfer of ITCs and GLs out of the mouth, as well as into and out of the stomach and intestinal compartments are first order rate reactions (Equations (6) and (7)).

(6) $$\frac{dC_{ITC\;i}}{dt}=k_{transfer\;\left(i-1\;to\;i\right)}\times \frac{V_{i-1}}{V_{i}}\times C_{ITC\;i-1}$$

and

(7) $$\frac{dC_{GL\;i}}{dt}=k_{transfer\;\left(i-1\;to\;i\right)}\times \frac{V_{i-1}}{V_{i}}\times C_{GL\;i-1}$$

The rate constants for transfer from the mouth, *k_mouth_*, for the stomach compartments, *k_SH_* and *S*, and intestinal compartments, *k_tsi_* and *k_tli_*, are the inverses of the residence time of each compartment (Table 1). Due to differences in some compartment volumes (V), volume ratios are considered.

The gut formation of ITCs, nitriles, and erucin are also represented by the following first order rate reactions,

(8) $$\frac{dC_{ITC}}{dt}=k_{f}\times C_{GL}$$

(9) $$\frac{dC_{Nitrile\;\&\;Erucin}}{dt}=k_{eni}\times C_{GL}$$

where *k_f_* is the rate constant of formation for ITC, and *k_eni_* is the rate constant of formation for erucin and nitrile. The change in GL concentration in the gut is proportional to the formation of ITC, nitrile, and erucin.

(10) $$\frac{dC_{GL}}{dt}=-(k_{f}\times C_{GL})-(k_{eni}\times C_{GL})$$

Absorption into the blood is defined by the following equation,

(11) $$\frac{dC_{ITC}}{dt}=k_{a}\times C_{ITC}$$

where *k_a_*, is the rate of absorption (passive diffusion) from the intestines and it is proportional to the effective permeability (*P_eff_*) of sulforaphane and inversely proportional to the radius, R, of the intestines [49].

(12) $$k_{a}=\frac{2P_{eff}}{R}$$

Each compartment is defined by mass balanced differential equations that contain the processes just described. For example, the full ITC and GL differential equations for the seventh small intestine compartment and first large intestine compartment are shown, Equations (13)–(16),

Seventh small intestine compartment:

(13) $$\begin{array}{cccc} \;\frac{dC_{ITC\;SI7}}{dt}=\frac{V_{SI6}}{V_{SI7}} & \times (k_{tSI}\times C_{ITC\;SI6}) & -(k_{tSI}\times C_{ITC\;SI7}) & -(k_{a}\times C_{ITC\;SI7})\\ & \mathrm{ITC In} & \mathrm{ITC Out} & \mathrm{ITC Absorption} \end{array}$$

(14) $$\begin{array}{ccc} \frac{dC_{GL\;SI7}}{dt}=\frac{V_{SI6}}{V_{SI7}} & \times (k_{tSI}\times C_{GL\;SI6}) & -(k_{tSI}\times C_{GL\;SI7})\\ & \mathrm{GL In} & \mathrm{GL Out} \end{array}$$

First large intestine compartment:

(15) $$\begin{array}{ccccc} \frac{dC_{ITC\;LI1}}{dt}=\frac{V_{SI7}}{V_{LI1}} & \times (k_{tSI}\times C_{ITC\;SI7}) & +(k_{f}\times C_{GL\;LI1}) & -(k_{tLI}\times C_{ITC\;LI1}) & -(k_{a}\times C_{ITC\;LI1})\\ & \mathrm{ITC In} & \mathrm{ITC Formation} & \mathrm{ITC Out} & \mathrm{ITC Out} \end{array}$$

(16) $$\begin{array}{ccccc} \frac{dC_{GL\;LI1}}{dt}=\frac{V_{SI7}}{V_{LI1}} & \times (k_{tSI}\times C_{GL\;SI7}) & -(k_{f}\times C_{GL\;LI1}) & -(k_{tLI}\times C_{GL\;LI1}) & -(k_{eni}\times C_{GL\;LI1})\\ & \mathrm{GL In} & \mathrm{ITC Formation} & \mathrm{GL Out} & \mathrm{Erucin/Nitrile Formation} \end{array}$$

Full list of differential equations for all compartments are in the Supplementary Files.

### 2.4. Simulink Model

The mathematical equations used to represent the different compartmental processes were translated to a block diagram on Matlab’s Simulink application (Matlab R2020a). Code scripts written on the MATLAB (MathWorks) interface integrated with the Simulink block diagram model to run sensitivity analyses and to perform fitting on the five different broccoli products.

### 2.5. Matlab Coding and Fittings

Sensitivity Analysis. Sensitivity analysis was performed to see the effect of changing parameter values on the model output. A parameter was changed within literature determined ranges while other parameters were kept constant. The analysis was performed for the different broccoli products, NoBF, LowBF, MedBF, HighBF, and HighBP.

Model Fittings. After the sensitivity analysis, parameters with the most influence on the simulation output, were used for fitting the model to each participant’s data set. The fitting was done using a Trust-Region-Reflective Least Squares algorithm from the least squares data fitting solver of Matlab’s Optimization Toolbox. The simulation period was 1600 min (26 h). Values of parameters used for fitting are in Table 1. This fitting procedure yielded parameter estimates that gave the best fit between the model simulations and the experimental data of the intervention study.

### 2.6. Statistical and Data Analysis

Confidence Intervals for each parameter estimate were determined at 90% confidence using Matlab’s non-linear parameter confidence interval function. This function also provided covariance matrices that were used to calculate the correlation coefficients.

Using Matlab’s trapezoidal numerical integration function, the cumulative SR excreted per participant for the experimental and predicted data sets were calculated.

Full codes are provided in the supplementary files.

## 3. Results

### 3.1. Sensitivity Analysis and Parameters Selection

The model’s sensitivity towards twelve parameters was tested. Based on the analysis, three parameters were used in model fittings for HighBP and HighBF, seven for Med and Low BF, and five for NoBF (Table 2).

**Figure 3:**
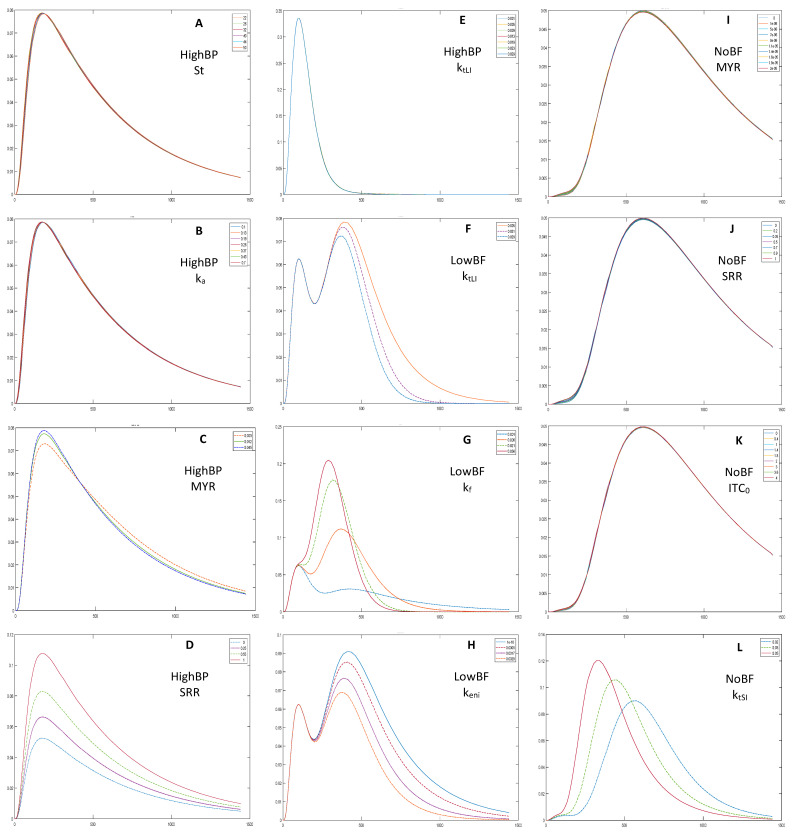
HighBP sensitivity analysis for (**A**) gastric emptying time, St; (**B**) the rate constant of absorption, k_a_; (**C**) myrosinase content, MYR; (**D**) the ratio of GR converted to SR in the mouth, SRR; (**E**) large intestine transit rate constant, k_tLI_. LowBF sensitivity analysis for (**F**) large intestine transit rate constant, k_tLI_; (**G**) ITC formation rate constant in the gut, k_f_; and (**H**) erucin and nitrile formation rate constant in the gut, k_eni_. NoBF sensitivity analysis for (**I**) myrosinase content, MYR; (**J**) the ratio of GR converted to SR in the mouth, SRR; (**K**) and initial ITC concentration (ITC_0_) and (**L**) small intestine transit rate constant, k_tSI_. The sensitivity analysis shows how the simulation output changes when all parameters are kept constant while one changes.

Parameters that did not affect model output were excluded. Gastric emptying time (St), and the rate constant of absorption (k_a_), were excluded from all model fittings because they had insignificant effects on the output (example in Figure 3A,B). As expected, myrosinase content (MYR), initial GR concentration (Cgl_0_), initial SR concentration (ITC_0_), and the ratio of GR converted to SR in the mouth (SRR), caused a direct and proportional upward shift to the HighBP, HighBF, MedBF, and LowBF simulation outputs (example in Figure 3C,D). The MYR and ITC_0_ contents were low for NoBF, therefore, of the broccoli product related parameters, only Cgl_0_ affected the output. Furthermore, the conversion ratio of GR to SR in the mouth (SRR), which depends on myrosinase content, did not affect the output of NoBF during the sensitivity analysis since myrosinase was inactive (Figure 3J). The first stomach rate constant (K_SH_), small intestine transit rate constant (k_tSI_) and SR elimination from the blood (k_e_), also caused proportional upward shifts but a narrowing of the curves was observed (example Figure 3L). The gut parameters, ITC formation (k_f_) and erucin and nitrile formation (k_eni_) rate constants, had opposite effects on outputs. Increases in k_eni_ resulted in downward shifts and narrowing of the output curves (Figure 3H), while the curves shifted upwards for k_f_ (Figure 3G). Increasing gut transit rate constant (k_tLI_), decreased the size of the second peak for MedBF and LowBF, and the single peak for NoBF (example in Figure 3F). Changes in k_tLI_ did not affect outputs of HighBP and HighBF products (Figure 3E).

### 3.2. Model Fittings

**Figure 4:**
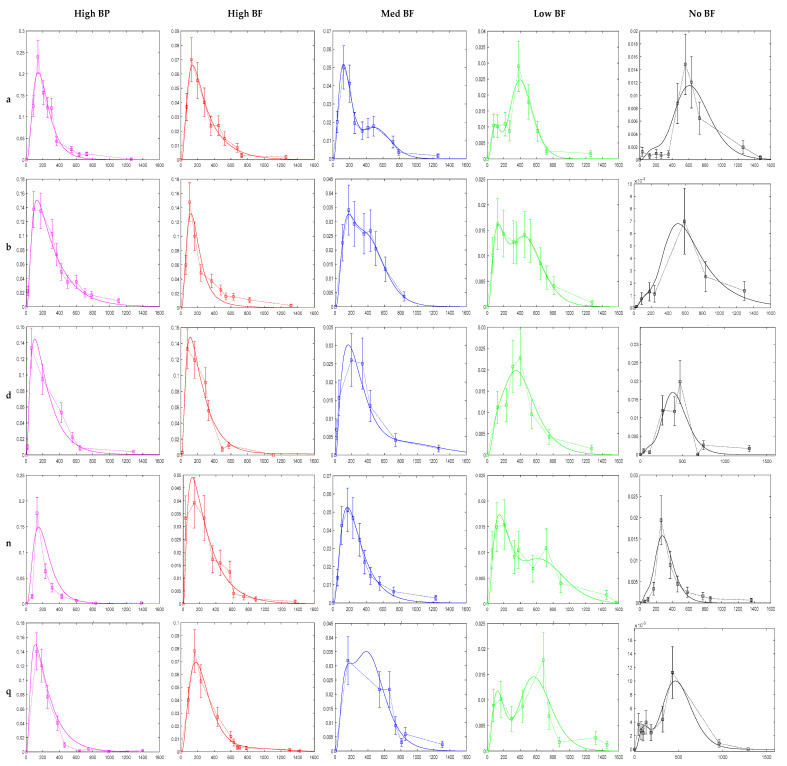
Model fittings for participants a, b, d, n, q. X and Y axis for each graph is, time (min) and sulforaphane conjugate excretion rate (µmol/min), respectively. Experimental data are the square bullets, and the solid lines are the model fits. Error bars represent potential experimental error from the analytical techniques used by Oliviero et al. to measure the amounts of ITC conjugates in urine. All participant model fittings are in Figure S1: Model Fittings Results for All Participants in the Supplementary Files.

The model was successfully fit to the data of each participant and for each product. Figure 4 shows the model fits for five of the participants and for all five broccoli products. 75 fittings were possible (15 participants times five products) but only 72 fitting were performed. Three data sets were excluded due to lack of data. While four data sets had to be preprocessed before fitting.

Few data sets had poor fits. Participant *m*, for the MedBF product, was underfitted while participants *q*, MedBF, and *g*, LowBF, were overfitted (Figure 4 and Figure S1: Model Fittings Results for All Participants in the Supplementary Files).

### 3.3. Bioavailability of Sulforaphane

Bioavailability was calculated by dividing the cumulative amounts of SR by the amount of GR in the broccoli products. Cumulative amounts of SR for the experimental data and model data were determined using Matlab’s trapezoid function to integrate. There were small differences between the predicted bioavailability and experimental bioavailability (Table 3). Average HighBP predictions were 2% larger than the calculated experimental bioavailability. The difference was 1% for MedBF, 0.9% for LowBF, and 0.1% for HighBF and NoBF.

### 3.4. Mouth and Gut Parameter Estimations

Table 4, Table 5 and Table 6 shows the distribution (boxplots) of mouth and gut parameter estimates for each product type. Interquartile ranges (IQR) for the parameter estimates varied widely. The distributions of most parameter estimates are positively skewed indicating that 50% of participants are less variable within the first two quartiles than the 50% of participants in quartile three and four.

SRR, the ratio of GR that gets converted to SR in the mouth, was estimated for each participant who consumed the HighBP, HighBF, Med and Low BF products (Table 4). SRR was not fitted for the NoBF product since the amount of MYR is very low. Therefore, any GR converted to SR in the mouth is insignificant for the NoBF product. The medians for HighBF, MedBF, and LowBF mean that half of the participants converted less than 19% GR to SR in the mouth. HighBP distribution, on the other hand, is negatively skewed with half the participants converting 35 to 60% of GR (median = 0.357) to SR.

K_f_ and k_eni_ represent the rate of formation of sulforaphane and erucin and nitriles, respectively, by gut bacteria in the large intestine. These parameters were estimated for the MedBF, LowBF and NoBF products but not for the High myrosinase products. K_f_ was fixed at 0.0033 min^−1^ and k_eni_ was fixed at 0.0015 min^−1^ for both products. All distributions are positively skewed indicating that at 50% of the participant metabolize GR to SR slower than 0.042 min^−1^ for MedBF, 0.012 min^−1^ for LowBF, and 0.003 min^−1^ for NoBF (Table 5); and they metabolize GR to erucin and nitrile slower than 0.017 min^−1^ for MedBF, 0.033 min^−1^ for LowBF, and 0.003 min^−1^ for NoBF (Table 6).

The results of other parameters are discussed in Appendix B.

### 3.5. Certainty of Parameter Estimates

For each participant and broccoli product, 90% confidence intervals were calculated to determine the range of parameters that are likely to include the parameter estimates. The confidence intervals were very large and are therefore not a good measure of certainty in the parameter estimates. The percentage of participants that had intervals larger than 30% on either side of the estimated parameter, was 62% of participants for HighBP, 53% for HighBF, 96% for MedBF, 98% for LowBF, and 100% for NoBF. The large confidence intervals were due to the limited data of each participant.

### 3.6. Goodness of Fit

**Figure 5:**
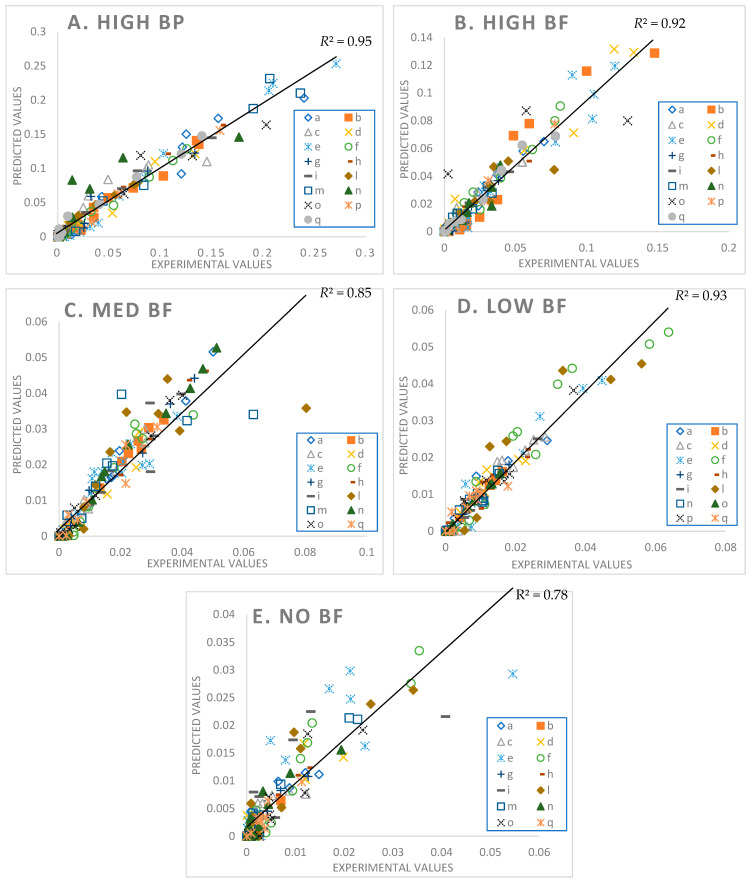
Parity plot of High MYR Broccoli Powder (**A**), High MYR Broccoli Florets (**B**), Medium MYR Broccoli Florets (**C**), Low MYR Broccoli Florets (**D**), and No MYR Broccoli Florets (**E**) with lumped participant data. Participants are designated by letters.

A comparison between the experimental and the predicted values are shown in Figure 5.

The best fits are for the HighBP product (*R*^2^ = 0.95), followed by LowBF (*R*^2^ = 0.93), HighBF (*R*^2^ = 0.92), MedBF (*R*^2^ = 0.85), and lastly NoBF (*R*^2^ = 0.78). Most predicted values for NoBF are higher than their corresponding experimental values (Figure 5E). Of the five broccoli products NoBF was the most difficult to fit due to the narrow peaks and wider base for most of the excretion curves compared to the other products (see NoBF simulation results in Figure S1: Model Fittings Results for All Participants in the supplementary files.). A noticeable feature of the model for the HighBF, MedBF, LowBF, and NoBF products (Figure 5B–E and Figure S1), is that the model solutions for some participants (e.g., participants e, l, n) approached excretion rates of 0 faster than the data points. The fit on the tail end of the data points, as well as the general fits for NoBF, may be improved by updating the model to include future physiological information on GR gut conversions, ITC absorption, distribution, and elimination.

## 4. Discussion

### 4.1. Sensitivity Analysis and Selection of Parameters

Sensitivity analysis for each product type was conducted to understand how changing one parameter and keeping the other constant would affect the model simulation output. Parameters to be estimated in model fittings were selected based on the results of the sensitivity analysis and preliminary model fittings. Given the low amount of data (6–15 data points) per participant, this procedure helped reduce the number of parameters fitted to the most necessary, thereby increasing the degrees of freedom.

Parameters that affected model output but were excluded from final fittings were k_e_, MYR, Cgl_0_, and ITC_0_. The model was sensitive to k_e_ for the HighBP, HighBF, MedBF, and LowBF products. However, k_e_ was not included in the fittings for HighBP and BF due to the poor model convergence. For all products, MYR and Cgl_0_ were fixed at the concentrations calculated from the Oliviero study (Table 1 and Matlab codes in the Supplementary Files). The initial concentration of SR (ITC_0_) calculated for HighBP, HighBF, and NoBF were kept at the values derived from the Oliviero study, while for MedBF and LowBF, ITC_0_ was reduced in order to get a good fit (Table 1). The reduction in ITC_0_ may be explained by the high variability in the measured amounts of SR in the broccoli products. Oliviero et al. [35] measured SR in triplicates and the standard deviation for MedBF was 28.4% of the average, while for LowBF, it was 64.3%; the standard deviations were the two highest out of the five products. ITC_0_ values used in the model were averages based on a sample size of three. Therefore, it is possible that most participants were consuming less SR initially. With the lower ITC_0_ values, the fittings for MedBF and LowBF had lower means squared error (MSE) values and better fits visually. As with k_e_, the model function appeared to be stuck in a local minimum when ITC_0_ was used at its original values. A better approach for future modeling might be to fit MYR, Cgl_0_, and ITC_0_ for all participants simultaneously. With the estimated MYR, Cgl_0_, and ITC_0_ kept constant, the remaining parameters sensitive to the model would be fitted.

More data are needed per participant, >15 data points, to get higher degrees of freedom. The number of data points per participant becomes important the greater the number of parameters being estimated. Obtaining a specified number of urine excretion data points from an intervention study is understandably not easy to achieve. Collecting blood samples during the intervention period in addition to urine samples would provide additional data for the model fittings that may improve parameter estimations as well as increase the number of parameters being estimated.

### 4.2. Model Fittings

The model succeeded in fitting the data of the various broccoli products well within the experimental error for most of the participants (Figure 4 and Figure S1). It also described the myrosinase mediated conversion of GR in the mouth and the microbial conversion of GR in the gut well. The quick appearance (within 2–3 h after consumption) of the single excretion peaks for HighBP and HighBF products, represents excretion rates of SR that were initially consumed or formed in the mouth, and were absorbed in the small intestine. The NoBF product, which had an insignificant amount of myrosinase, had single peaks that appeared much later (7–8 h after consumption). The NoBF peaks represent excretion rates of SR that was formed by gut bacteria and absorbed only in the large intestine. MedBF and LowBF curves tend to have two peaks. The first peak representing absorption from the small intestine and the second peak from the large intestine. This excretion pattern due to differences in myrosinase content have been observed by other authors [35,36,50,51].

### 4.3. Bioavailability of Sulforaphane

This compartment model is a good predictor of bioavailability. Other researchers observed that using a compartmental absorption and transit (CAT) model was better at predicting bioavailability than a single compartment model [52].

### 4.4. Mouth and Gut Parameter Estimations

The parameter estimates clearly show the variability between individuals in the amount of GR converted to SR during mastication and in their gut bacteria activity. The distributions (Table 4, Table 5 and Table 6) also vary across product categories. The median GR conversion ratio, SRR, is higher for HighBP compared to the HighBF, MedBF, and LowBF broccoli products. 50% of the HighBP participants have conversion ratios similar to what Sarvan et al. [20] found in their study for 0.5 min and 1 min cooked broccoli, where after chewing, 41% and 60% GR was converted to SR. The fact that the overall distribution for HighBP is shifted to higher values compared to HighBF demonstrates the importance of the product matrix. Although, both had the same myrosinase content, most of the participants could convert more GR to SR after consuming the powder. General differences in chewing patterns between individuals have been documented [23]. However, more specific studies on the effect of chewing patterns correlated to conversion GR ratios may provide insights into the variations observed between individuals in the parameter distributions.

The variation between products is significant for the gut parameters, k_f_ and k_eni_. The IQR for LowBF and NoBF were expected to be similar since most of the myrosianse was inactivated in both products. However, the NoBF IQR was significantly smaller, which implied that the participants in the Oliviero study had very similar gut bacteria or that their overall bacterial activity was similar. Unfortunately, data on the gut microbial population of the participants was not available to correlate to the parameter estimation results. It is well known that gut bacterial populations differ between individuals and populations [7,53,54,55]. Forty-seven bacterial species having been identified as having GL metabolizing activities in-vitro [9], but only a few have been investigated for their GL bioconversion mechanisms: *Enterobacter cloacae* [56], *Lactobacillus agilis R16* and *Escherichia coli VL8* [57], *Bacteroides thetaiotaomicorn* [58].

## 5. Conclusions

A physiological-based multicompartment digestion and absorption model, was developed to describe the kinetics and bioavailability of sulforaphane (SR) from broccoli, and to evaluate how the derived parameters are impacted by inter-individual variation. The model included reactions during digestion in the mouth and gut. It successfully fit participant data and was able to describe bioavailability of SR very well as there were minimal differences between the predicted and experimentally bioavailability. The parameters estimated during the model fitting represented physiological aspects of the digestion process, which were also sources of inter-individual variability. For the digestion of broccoli, the parameters that represented sources for variation between individuals were SRR, the ratio of GR converted to SR during mastication, and *k_f_* and *k_eni_*, the conversion rate constants of GR to SR or other break down products. The inter-individual variability between participants was captured in the variability of some these estimates. However, it was not possible to correlate the variability between participants to specific physical attributes, such as chewing patterns or predominant gut microbes, as that information for the participants was not available.

The model’s predicted values fit the experimental values very well, especially for the high and low myrosinase products. The lower quality of the fit for the no myrosinase product, indicates the need to improve the model’s representation of microbial gut conversions. The work completed in this study is a preliminary step in creating a validated model, which, in the future, could be a useful tool in being able to predict the biological effects of SR and possibly other bioactive compounds. A future predictive model has the potential to positively influence the growing field of personalized nutrition.

## Figures and Tables

**Table 2:** Summary of parameters that influenced the simulation outputs for each broccoli product during the sensitivity analysis. Parameters with a check mark (✓) were used for model fittings; parameters with an O, influenced simulation output but were not used in model fittings; parameters with an x did not affect output during the sensitivity analysis and were excluded from fitting. Three parameters were used to fit HighBP and HighBF, seven for MedBF and LowBF, and five for NoBF.

	k_SH_	SRR	k_f_	k_e_	k_tSI_	k_tLI_	k_eni_	Cgl_0_	ITC_0_	MYR	k_a_	St
HighBP	✓	✓	x	o	✓	x	x	o	o	o	x	x
HighBF	✓	✓	x	o	✓	x	x	o	o	o	x	x
MedBF	✓	✓	✓	✓	✓	✓	✓	o	o	o	x	x
LowBF	✓	✓	✓	✓	✓	✓	✓	o	o	o	x	x
NoBF	✓	x	✓	x	✓	✓	✓	o	o	o	x	x

**Table 3:** Average sulforaphane (SR) bioavailability values ±SD for the experimental data and model. There were minor differences between the experimental bioavailability and model bioavailability for each broccoli product type.

	Average SR Bioavailability (%)		
	Experimental Data	Model	Difference
HighBP	63 ± 0.2	65 ± 0.1	2%
HighBF	33 ± 0.1	33 ± 0.1	0.1%
MedBF	25 ± 0.1	24 ± 0.1	1%
LowBF	19 ± 0.1	18 ± 0.1	0.9%
NoBF	10 ± 0.04	10 ± 0.04	0.1%

**Table 4:** SRR estimation results (horizontal red bar: median, blue box second and third quartile, whiskers first and fourth quartiles).

High BP (*n* = 15)	High BF (*n* = 15)	Med BF (*n* = 14)	Low BF (*n* = 14)	No BF
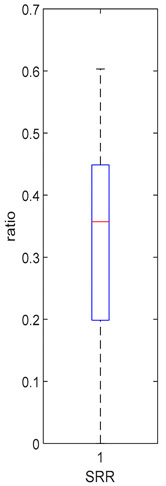	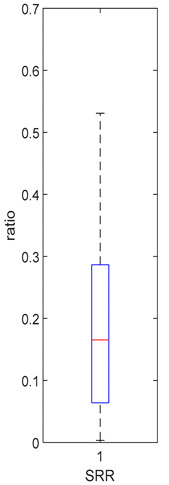	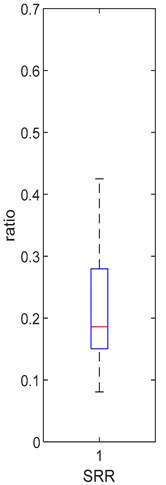	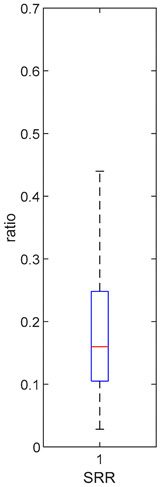	----
Median: 0.357 IQR: 0.251 IQR/Med: 0.7 Outliers: None	Median: 0.165 IQR: 0.223 IQR/Med: 1.4 Outliers: None	Median: 0.186 IQR: 0.13 IQR/Med: 0.7 Outliers: None	Median: 0.160 IQR: 0.143 IQR/Med: 0.9 Outliers: None	SRR parameter not fitted. Fixed at 0.047.

**Table 5:** k_f_ estimation results (horizontal red bar: median, blue box second and third quartile, whiskers first and fourth quartiles, +: outliers).

High BP(*n* = 15)	High BF(*n* = 15)	Med BF(*n* = 14) *	Low BF(*n* = 14) *	No BF(*n* = 14) *
----	----	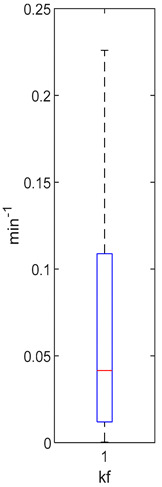	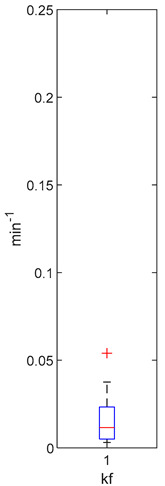	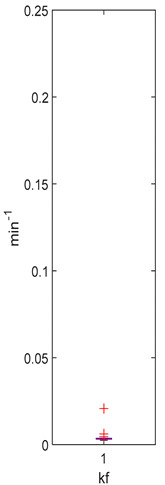
Not fitted. k_f_ fixed at 0.0033 min^−1^.	Not fitted. k_f_ fixed at 0.0033 min^−1^.	Median: 0.042 min^−1^ IQR: 0.097 min^−1^ IQR/Med: 2.3 Outliers: c (0.555 min^−1^)	Median: 0.012 min^−1^ IQR: 0.018 min^−1^ IQR/Med: 1.5 Outliers: *n* (0.054 min^−1^)	Median: 0.003 min^−1^ IQR: 0.001 min^−1^ IQR/Med: 0.3 Outliers: g (0.006 min^−1^) h (0.005 min^−1^) q (0.021 min^−1^)

* Some or all outliers excluded for better visual presentation and comparison of boxplots. Outliers are values more than 1.5 times the IQR. See Supplementary Files for plotted outliers and for expanded view of the NoBF k_f_ boxplot.

**Table 6:** k_eni_ estimation results (horizontal red bar: median, blue box second and third quartile, whiskers first and fourth quartiles, +: outliers).

High BP(*n* = 15)	High BF(*n* = 15)	Med BF(*n* = 14) *	Low BF(*n* = 14)	No BF(*n* = 14)
----	----	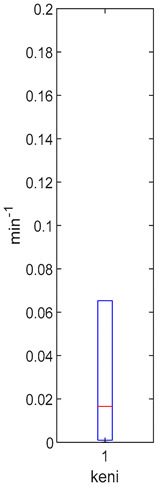	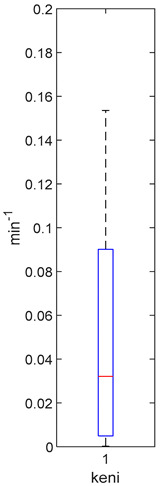	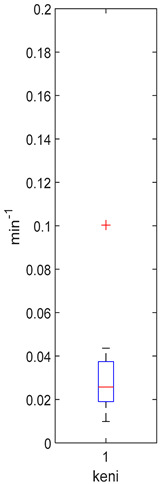
Not fitted. k_eni_ fixed at 0.0015 min^−1^.	Not fitted. k_eni_ fixed at 0.0015 min^−1^.	Median: 0.017 min^−1^ IQR: 0.064 min^−1^ IQR/Med: 3.8 Outliers: c (1.95 min^−1^) h (0.576 min^−1^) *n* (0.26995 min^−1^)	Median: 0.033 min^−1^ IQR: 0.085 min^−1^ IQR/Med: 2.6 Outliers: None	Median: 0.026 min^−1^ IQR: 0.018 min^−1^ IQR/Med: 0.7 Outliers: g (0.100 min^−1^)

* Some or all outliers excluded for better visual presentation and comparison of boxplots. Outliers are values more than 1.5 times the IQR. See Supplementary Files for plotted outliers.

## Data Availability

Not applicable.

## Supplementary Materials

The following are available online at https://www.mdpi.com/article/10.3390/foods10112761/s1. Table S1: Compartment Equations, Tables S2–S8: Parameter Box Plots, Figure S1: Model Fittings Results for All Participants, Matlab Codes and Simulink Model

## Appendix A. Myrosinase Calculations

The amount of myrosinase (mg MYR/mg Broccoli) was calculated based on the methods of Oliviero et al. [35] and the research of Roman et al. [42] Oliviero et al. used a spectrophotometric method to determine the myrosinase activity in the different broccoli products. Roman et al. investigated two mechanisms for substrate inhibition during the conversion of GLs to ITCs by modeling their experimental results (enzyme reaction rate vs. sinigrin concentration) using the modified Michaelis–Menten *K_i_* = netics with Substrate Inhibition (MMSI) model (Equation (A1)).

(A1) $$v=\frac{V_{max}\cdot \left[S\right]}{K_{m}+\left[S\right]+\frac{{\left[S\right]}^{2}}{K_{i}}}$$

*V_max_* (µM/min*mg MYR) is the maximum rate of the system, *K_m_* (µM) is the Michaelis-Menten constant, *K_i_* (µM) is the inhibition constant, *S* is the substrate concentration, and *v* is the reaction rate. This physiological-based model assumes substrate inhibition occurs in the catalytic site.

*Part I. Steps to determining myrosinase content in each broccoli product.*

1. Experimental data points were extracted from the reaction rate vs. sinigrin concentration graph [42].

**Sinigrin Concentration (µM)**	**Initial Reaction Rate (µmol/min)**
5	0.03
10	0.05
25	0.16
40	0.24
50	0.29
75	0.42
100	0.66
149	0.58
199	0.59
248	0.57
298	0.52

2. The MMSI equation (Equation (A1)) was used to model the data. The parameters, *V_max_*, *K_m_*, and *K_i_*, were solved by minimizing the sum of squares difference using Excel’s SUMXMY2 function and solver.

**Parameter Estimates**	
*V_max_*	0.96 µM/min
*K_m_*	86.51 µM
*K_i_*	780.05 µM

3. The specific enzyme activity of Myrosinase was calculated using information from the materials Oliviero et al. used to determine activity and the parameter estimates from step 2.

Concentration of sinigrin in reaction mixture used by Oliviero et al. to determine myrosinase activity was calculated as follows:

$$\frac{30\;\mathrm{mg}}{\mathrm{mL}}\times \frac{\mathrm{mol}}{397.5\;g}\times \frac{g}{1000\;\mathrm{mg}}\times 10^{6}=75.5\;\frac{\mathrm{umol}}{\mathrm{mL}}$$

$$\begin{array}{c} 75.5\;\frac{\mu \mathrm{mol}}{\mathrm{mL}}\times \frac{0.05\;\mathrm{mL}\;\sin\mathrm{igrin}\;\mathrm{solution}}{1.105\;\mathrm{mL}}\times 1000\frac{\mathrm{mL}}{L}\\ =3415\;\mathrm{uM}\;\mathrm{Sinigrin}\;\mathrm{in}\;\mathrm{reaction}\;\mathrm{mixture} \end{array}$$

Equation (A1) was used to calculate specific enzyme activity

$$\frac{\left(0.96\frac{\mu M}{\min*\mathrm{mg}\;\mathrm{MYR}}\right)\times 3415\;\mu M}{86.51\;\mu M+3415\;\mu M+\frac{{\left(3415\;\mu M\right)}^{2}}{780.05\;\mu M}}=0.178\frac{\mathrm{umol}}{\mathrm{mg}\;\mathrm{MYR}*\min}$$

4. The Oliviero *MYR* activity (column A below) for each product type was divided by 5000mg to determine the µmol MYR/mg broccoli * min (column B). Column B was divided by the specific enzyme activity (0.178 µmol/mg MYR*min) to obtain the mg MYR/mg Broccoli (column C).

	**A**	**B**	**C**
	**MYR Activity** **(Units/5 g dry wt Broccoli)**	**µmol/mg Broccoli * min**	**mg MYR/mg Broccoli**
High MYR BP	31	0.0062	0.035
High MYR BF	31	0.0062	0.035
Medium MYR BF	5.8	0.00116	0.007
Low MYR BF	0.5	0.0001	0.000563
No MYR BF (<0.01)	0.01	0.000002	0.0000113

*Part II. Estimating V_max_, K_m_, and K_i_, for the myrosinase conversion of GR in the mouth*

1. Experimental data points were extracted from the reaction rate vs. glucoraphanin concentration graph [42].

**Glucoraphanin Concentration (µM)**	**Initial Reaction Rate (µmol/min)**
5	0.02
8	0.09
10	0.11
25	0.25
50	0.45
75	0.7
90	1.14
100	1.33
150	1.25
200	1.19
250	1.18
300	1.05

2. The MMSI equation (Equation (A1)) was used to model the data. The parameters, *V_max_*, *K_m_*, and *K_i_*, were solved by minimizing the sum of squares difference using Excel’s SUMXMY2 function and solver.

**Parameter Estimates**	
*V_max_*	2070 µmol/min
*K_m_*	110.16 µM
*K_i_*	893.02 µM

## Appendix B. Parameter Estimates Results and Discussion for K_SH_, K_tSI_, K_tLI_, and K_e_

**Table A1:** K_SH_ estimation results.

High BP(*n* = 15) *	High BF(*n* = 15) *	Med BF(*n* = 14) *	Low BF(*n* = 14)	No BF(*n* = 14)

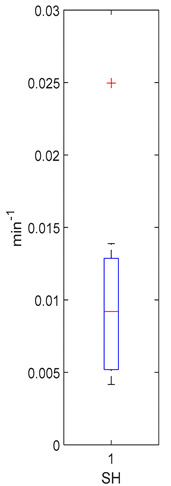
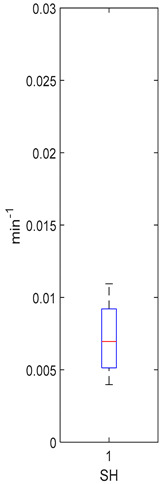
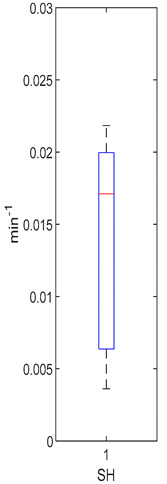
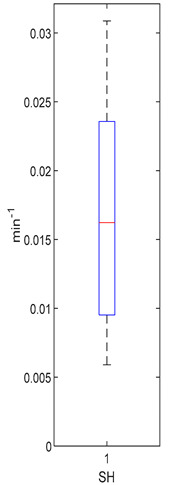
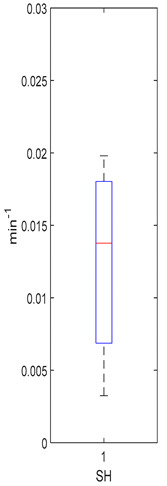
				
Median: 0.009 min^−1^ IQR: 0.008 min^−1^ IQR/Med: 0.9 Outliers: d (9.408 min^−1^) l (0.752 min^−1^) *p* (0.025min^−1^)	Median: 0.007 min^−1^ IQR: 0.004 min^−1^ IQR/Med: 0.6 Outliers: d (0.105 min^−1^)	Median: 0.017 min^−1^ IQR: 0.014 min^−1^ IQR/Med: 0.8 Outliers: d (0.181 min^−1^)	Median: 0.016 min^−1^ IQR: 0.014 min^−1^ IQR/Med: 0.9 Outliers: None	Median: 0.014 min^−1^ IQR: 0.011 min^−1^ IQR/Med: 0.8 Outliers: None

* Some or all outliers excluded for better visual presentation and comparison of boxplots. Outliers (+) are values more than 1.5 times the IQR. See supplementary tables for plotted outliers.

Stomach emptying was described as biphasic by having two stomach compartments. The first stomach compartment was for the delay caused by the disintegration of large food particles before it is emptied into the duodenum. Delayed emptying has been observed for wheat products [59] and other solid foods [31,39]. Since participants ate a 30-g raisin bun, right after ingesting 5 g of broccoli florets, a compartment was included to represent disintegration of both food products. K_SH_, the rate constant from the first stomach compartment to the second, is the inverse of the time required for food disintegration. Parameter estimates for K_SH_ are shown in Table A1. Excluding the outliers, HighBP (IQR = 0.008 min^−1^) and HighBF (IQR = 0.004 min^−1^), have normal distributions compared to Med, Low and NoBF products. Comparatively, the median values of HighBP (0.009 min^−1^) and High BF (0.007 min^−1^) are smaller than the medians of the MedBF (0.017 min^−1^), LowBF (0.016 min^−1^), and NoBF (0.014 min^−1^) products. These values imply that most participants who consumed MedBF, LowBF and NoBF, required less time for stomach disintegration of the broccoli and the raisin bun than HighBF and HighBP. Since all participants consumed the same amounts of florets and raisin bun, differences in food disintegration were only expected between individuals due to physiological differences. However, the median and distribution differences between the High MYR products and the remaining three were not expected. Kong et al. [60] observed longer disintegration times in their stomach model for raw and 2 min cooked carrots versus their 6 min cooked carrots. Since the high MYR broccoli florets, HighBF, were less heat processed, a harder product texture may be the cause of its longer disintegration time. However, this does not explain why the powdered product, HighBP does not have shorter disintegration times compared to the MedBF, LowBF, and NoBF. It is possible that the raisin bun consumed at the time participants consumed HighBP was harder in texture than when the other broccoli products were consumed.

**Table A2:** k_tSI_ estimation results.

High BP(*n* = 15) *	High BF(*n* = 15) *	Med BF(*n* = 14) *	Low BF(*n* = 14)	No BF(*n* = 14)

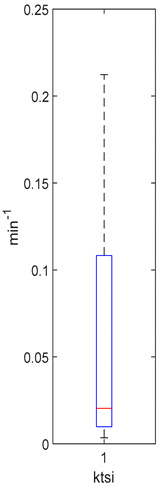
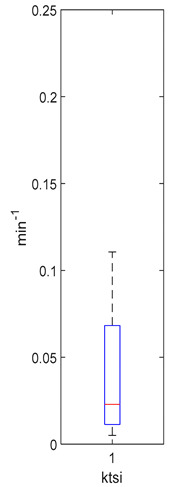
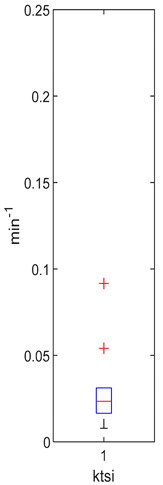
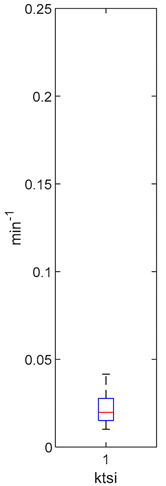
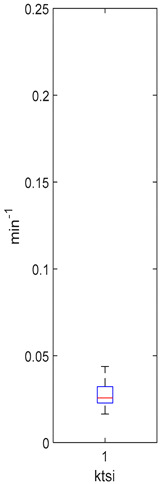
				
Median: 0.020 min^−1^ IQR: 0.098 min^−1^ IQR/Med: 4.9 Outliers: c (5.192 min^−1^)	Median: 0.023 min^−1^ IQR: 0.057 min^−1^ IQR/Med: 2.5 Outliers: n (1.369 min^−1^)	Median: 0.023 min^−1^ IQR: 0.014 min^−1^ IQR/Med: 0.6 Outliers: h (0.366 min^−1^) i (0.092 min^−1^) o (0.054 min^−1^)	Median: 0.020 min^−1^ IQR: 0.013 min^−1^ IQR/Med: 0.7 Outliers: none	Median: 0.026 min^−1^ IQR: 0.009 min^−1^ IQR/Med: 0.3 Outliers: none

* Some or all outliers excluded for better visual presentation and comparison of boxplots. Outliers (+) are values more than 1.5 times the IQR. See supplementary tables for plotted outliers.

**Table A3:** k_tLI_ estimation results.

High BP(*n* = 15)	High BF(*n* = 15)	Med BF(*n* = 14) *	Low BF(*n* = 14) *	No BF(*n* = 14) *
----	----			
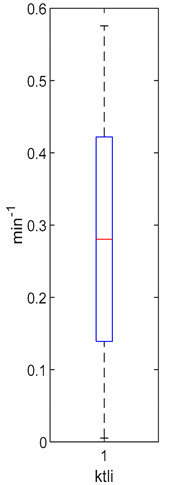
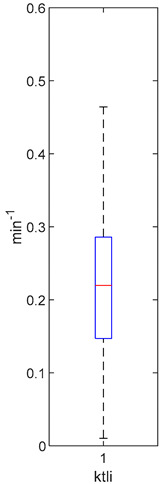
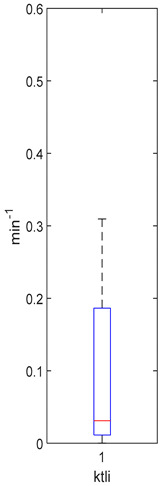
Not fitted. K_tLI_ fixed at 0.003 min^−1^.	Not fitted. K_tLI_ fixed at 0.003 min^−1^.	Median: 0.280 min^−1^ IQR: 0.284 min^−1^ IQR/Med: 1.0 Outliers: h (0.96575 min^−1^) i (0.92453 min^−1^)	Median: 0.220 min^−1^ IQR: 0.139 min^−1^ IQR/Med: 0.6 Outliers: *n* (0.9998 min^−1^)	Median: 0.031 min^−1^ IQR: 0.175 min^−1^ IQR/Med: 5.6 Outliers: q (1.0385 min^−1^)

* Some or all outliers excluded for better visual presentation and comparison of boxplots. Outliers (+) are values more than 1.5 times the IQR. See supplementary tables for plotted outliers.

The transit rate constants for each compartment of the small and large intestine are k_tSI_ and k_tLI_, respectively. The rate constants were determined based on the measured time it takes for food contents to transit through the small and large intestines. Excluding outliers, the distribution of k_tSI_ parameters for MedBF (IQR = 0.014 min^−1^), LowBF (IQR = 0.013 min^−1^), and NoBF (IQR = 0.009 min^−1^) are less variable compared to the High BP (IQR = 0.098 min^−1^) and High BF (IQR = 0.057 min^−1^) broccoli products (Table A2). Except for MedBF (outliers excluded), the parameter distributions for the four products are positively skewed. The median values for all broccoli products are similar, around 0.02 min^−1^, which is within the range of literature cited values—0.01228 min^−1^–0.2333 min^−1^ [26]. Across all products transit through the small intestine compartments takes 50 min (1/0.02 min^−1^) or longer for 50% of the participants. For each product, the percentage of k_tSI_ parameter estimates that fall within the literature cited range are: 100% NoBF, 93% LowBF, 71% MedBF, 67% HighBF, and 73% HighBP. K_tLI_ was not fitted for the HighBP and HighBF; it was fixed at 0.003 min^−1^ for both products (Table A3) because sensitivity analysis showed this parameter did not have an influence on the model’s output. The medians for MedBF (0.280 min^−1^), LowBF (0.220 min^−1^), and NoBF (0.031 min^−1^) are larger than literature ranges (0.002 min^−1^–0.003 min^−1^) for gut intestinal transit [61]. The parameter distributions are variable especially NoBF in which the interquartile range is 560% of the median. Furthermore, the distribution for NoBF is positively skewed, indicating that 50% of the participants had colon transit times longer than 32 min (1/0.031 min^−1^) per compartment or 3.8 h for the colon. Based on the skewness, the participants with the long transit times are not as variable as the 50% of participants with transit times shorter than 32 min per compartment. Nine out of the 15 participants in the Oliviero study were women and it is known that women have longer intestinal transit times compared to men [62]. The gender of the participants with their corresponding data were not provided, so it is impossible to correlate transit times to gender.

**Table A4:** k_e_ estimation results.

High BP(*n* = 15)	High BF(*n* = 15)	Med BF(*n* = 14) *	Low BF(*n* = 14)	No B(*n* = 14) *
----	----			
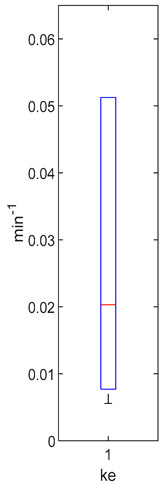
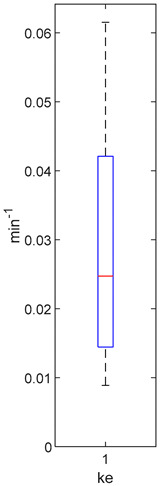
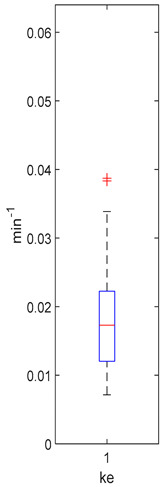
Not fitted. k_e_ fixed at 0.024 min^−1^.	Not fitted. k_e_ fixed at 0.024 min^−1^.	Median: 0.020 min^−1^ IQR: 0.043 min^−1^ IQR/Med: 2.2 Outliers: c (0.277 min^−1^) g (0.319 min^−1^) o (0.295 min^−1^)	Median: 0.025 min^−1^ IQR: 0.028 min^−1^ IQR/Med: 1.1 Outliers: None	Median: 0.017 min^−1^ IQR: 0.01 min^−1^ IQR/Med: 0.6 Outliers: f (0.038 min^−1^) g (0.039 min^−1^)

* Some or all outliers excluded for better visual presentation and comparison of boxplots. Outliers (+) are values more than 1.5 times the IQR. See supplementary tables for plotted outliers.

The rate constant for sulforaphane elimination from the blood, k_e_, was not fitted for HighBP and HighBF products, but rather fixed at 0.024 min^−1^ (Table A4). The median rates of elimination for MedBF, LowBF and NoBF are 0.020 min^−1^, 0.025 min^−1^, and 0.017 min^−1^, respectively. The IQR is largest for MedBF (0.043 min^−1^), followed by LowBF (0.028 min^−1^) and NoBF (0.01 min^−1^). Vermeulen et al. [36] fitted the plasma concentration data to one compartmental model and determined the elimination half-lives (t_0.5_) of SR for cooked and raw broccoli to be 4.6 h and 3.8 h, respectively. From half-lives (t_0.5_ = 0.693/ke), the elimination rate constants were calculated to be 0.002511 min^−1^ (cooked) and 0.003039 min^−1^ (raw) [36]. Vermeulen’s values are lower than all estimated parameters of MedBF, LowBF, and NoBF products. The difference is most likely due to the method used in determining k_e_, because Vermeulen et al. uses a more empirical model than the one described in this thesis.
